# Unintended consequences of programmatic changes to infant and young child feeding practices in Bangladesh

**DOI:** 10.1111/mcn.13077

**Published:** 2020-10-16

**Authors:** Md. Tariqujjaman, Mahfuzur Rahman, Sharmin Khan Luies, Gobinda Karmakar, Tahmeed Ahmed, Haribondhu Sarma

**Affiliations:** ^1^ Nutrition and Clinical Services Division International Centre for Diarrhoeal Disease Research, Bangladesh Dhaka Bangladesh; ^2^ Research School of Population Health The Australian National University Acton Australia

**Keywords:** Bangladesh, community health workers, infant and young child feeding, micronutrient powder, unintended consequences

## Abstract

BRAC, an international development organization, implemented a home‐fortification programme from 2014 to 2018 in Bangladesh. This study aimed to understand the unintended consequences of programmatic changes that occurred during the implementation of the programme on the prevalence of good infant and young child feeding (IYCF) practices and other associated factors. We used pooled data from eight cross‐sectional surveys and data from a series of qualitative investigations carried out as part of a mixed‐methods evaluation approach. A total of 6,479 caregivers of children aged 6 to 23 months participated in the surveys. The prevalence of good IYCF practices increased from baseline (42.1%) to midline (45.3%), but it decreased at the endline survey (31.9%). Qualitative investigations identified several reasons for low IYCF practices at the programme level, such as the withdrawal of community health worker (CHW) incentives for promoting IYCF, providing incentives for the home‐fortification of micronutrient powder (MNP) and changing the focus from IYCF promotion to MNP promotion. A multivariable generalized estimating equation model for pooled data revealed that caregivers were 28% (adjusted risk ratio [ARR]: 0.72, 95% CI [0.67, 0.78]) less likely to maintain good IYCF practices during the period when CHWs were not incentivized to promote IYCF compared to the period when CHWs were incentivized to promote it. The prevalence of good IYCF practices decreased from both baseline and midline to the endline survey due to the unintended consequences of the programmatic changes. An integrated intervention strategy to promote the home‐fortification of MNP and IYCF could be helpful to avoid unintended negative consequences of programmatic changes.

**Key messages**

- The prevalence of good IYCF practices increased from baseline (42.1%) to midline (45.3%), but it decreased in the endline survey (31.9%) due to the unintended consequences of programmatic changes such as withdrawal of community health worker (CHW) incentives for promoting IYCF practices.
- Household visits by CHWs and effective coverage of MNP had a positive association with good IYCF practices.
- When designing and implementing a programme, careful measures should be taken to avoid unintended negative consequences, and the integration of MNP interventions and CHW home visits could achieve the optimal IYCF practices.

## 1 INTRODUCTION

Suboptimal infant and young child feeding (IYCF) practices are a major contributor to undernutrition and related morbidity and mortality in children under the age of 5 years (Black et al., 2008). Nutritional deficiencies also increase the risk of mortality, morbidity and long‐term developmental delays (Black et al., 2013). According to the World Health Organization (WHO), exclusive breastfeeding for the first 6 months of life with early initiation, continuation of breastfeeding for 2 years and nutritionally adequate, safe and age‐appropriate complementary feeding starting at 6 months are recommended as appropriate IYCF practices (WHO, 2008). Recent analyses have found that, during the first 2 years of life, faltering growth is more severe; therefore, this time period is considered the critical ‘window of opportunity’ for improving child growth (Victora, de Onis, Hallal, Blossner, & Shrimpton, 2010).

Globally, approximately 35% of children aged 6 months are exclusively breastfed (Egata, Berhane, & Worku, 2013); 60% of children aged 6 to 8 months receive solid, semisolid and soft foods; and 58% of children continue breastfeeding up to the age of 2 years (United Nations Children's Fund, 2013), indicating suboptimal global IYCF practices. In South Asia, approximately 37% of children are exclusively breastfed; approximately 56% of children aged 6 to 8 months are fed solid, semisolid and soft foods, and only approximately 37% of children aged 6 to 23 months meet the requirements of a minimum acceptable diet (Senarath et al., 2012; United Nations Children's Fund, 2013). According to the Bangladesh Demographic and Health Survey (BDHS) 2014, 23% of caregivers of children aged 6 to 23 months maintained adequate IYCF practices, indicating suboptimal IYCF practices among most Bangladeshi caregivers (National Institute of Population Research and Training, 2014).

To improve IYCF practices globally, several interventions, such as integrated child development services provided through community‐based workers (Chaturvedi, Nakkeeran, Doshi, Patel, & Bhagwat, 2014), peer counselling by mother support groups (Kushwaha et al., 2014) and social and behavioural change communication (Menon et al., 2016), have been implemented. To improve IYCF practices and achieve the sustainable development goals related to child nutrition which are to obtain ‘zero hunger, achieve food security and improved nutrition and promote sustainable agriculture’ (goal 2), government and nongovernmental organizations (NGOs) in Bangladesh are providing several nutrition‐specific interventions such as promoting, protecting and supporting IYCF practices, controlling micronutrient deficiencies, managing moderate and severe acute malnutrition, providing nutrition services in emergencies and engaging in social and behaviour change communication on nutrition and nutrition‐sensitive interventions including food safety programmes, deworming programmes, water, sanitation and hygiene programmes at the community level (Plan & Nutrition, 2017). Intervention activities include counselling for women with children on exclusive breastfeeding, complementary foods and other related messages. To promote good IYCF practices, the National Nutrition Service provides training (through a standard training module based on the National Strategy for IYCF) to relevant service providers (Plan & Nutrition, 2017).

Despite having different intervention designs, many studies have observed fluctuations (increasing or decreasing patterns over time) in IYCF practices (Agho et al., 2016; Aguayo, 2017; WHO, 2018; Muhammad Hanif, 2013; Na, Aguayo, Arimond, Narayan, & Stewart, 2018; Nguyen et al., 2011). Some studies have indicated decreasing patterns of optimal IYCF practices (Na et al., 2018; Nguyen et al., 2011), and others have indicated increasing patterns of IYCF practices over time (Agho et al., 2016; Aguayo, 2017; WHO, 2018; Muhammad Hanif, 2013). The reasons for decreasing IYCF practices include cultural beliefs among caregivers, lack of information about IYCF practices, poor parental education and household poverty (Na et al., 2018; Nguyen et al., 2011). On the other hand, information, education and counselling provided by primary care workers/community resource persons by multiple contacts and community mobilization by effective behavioural change communication are the reasons for increasing IYCF practices (Agho et al., 2016; Aguayo, 2017; WHO, 2018). Moreover, there are several reasons for fluctuations in IYCF practices, including inadequate knowledge of healthcare providers, geographical location, degree of exposure to mass media, maternal age, child age and the utilization of antenatal and postnatal visits (Gautam, Adhikari, Khatri, & Devkota, 2016; Kabir et al., 2012; Kassa, Meshesha, Haji, & Ebrahim, 2016; Khanal, Sauer, & Zhao, 2013; Dibley et al., 2010; Na et al., 2018; Nguyen et al., 2011; Mihrshahi et al., 2010; Senarath, Agho, et al., 2012; Senarath, Godakandage, Jayawickrama, Siriwardena, & Dibley, 2012). In addition to these factors, the unintended consequences of programmatic changes can also play a significant role in fluctuations in IYCF practices (Vossenaar et al., 2017). Unintended consequences (negative or positive) of programmatic changes are an outcome that may be anticipated or unanticipated as a result of specific actions taken during the implementation of a programme. Findings from 11 MNP programmes undertaken in different geographic settings showed unintended negative consequences for IYCF practices, such as introducing complementary feeding before the child reaches 6 months of age, not purchasing or giving foods of animal sources to children when introducing MNP (the perception that MNP contains foods from animal sources) and the forced feeding of children (Siekmans, Bégin, Situma, & Kupka, 2017).

It is important to understand the unintended consequences of programmatic changes on IYCF practices and the factors associated with good IYCF practices so that the programme can be informed about the measures that should be taken to avoid negative unintended consequences. Previous literature suggests that there might be some unintended consequences (either positive or negative) during the implementation of a programme (Harris & Ogbonna, 2002; Rottenberg, 1991; Uddin, Sarma, Bari, & Koehlmoos, 2013) that should be taken into consideration. In the Maternal, Infant and Young Child Nutrition (MIYCN) programme, we did not suspect negative consequences, but after the endline survey, we found a significant decrease in good IYCF practices. Therefore, we aimed to explore the unintended consequences of the programmatic changes on good IYCF practices among caregivers of 6‐ to 23‐month‐old children and the factors associated with good IYCF practices.

## 2 METHODS

### 2.1 Study design and setting

The study used a multimethod evaluation with both qualitative and quantitative approaches. In quantitative analyses, we used pooled data from eight cross‐sectional surveys conducted at the household level, and these surveys were conducted according to a pre–post design as part of the evaluation of BRAC's MIYCN Phase 2 programme in Bangladesh. The surveys were conducted at different time points in the MIYCN programme areas of BRAC during the implementation period (Figure S1). Because the intervention of the MIYCN programme was rolled out in different phases, the first baseline survey and the corresponding midline and endline surveys were conducted in September 2014 to 2017 among 10 districts in the northern part of Bangladesh. The second baseline survey and the corresponding midline and endline surveys were conducted in March–April 2015 to 2018 in 15 districts in the eastern part of the country. Finally, the third baseline survey and the corresponding endline survey were conducted in April–May 2016 to 2018 in nine districts of Bangladesh. The evaluation team had to forgo the midline survey for Phase 3 in consensus with the implementers and collaborators because, after 2 years of implementation, the programme was considered mature, and therefore, an endline survey of the last phase (Phase 3) was done in place of the midline survey. The surveys were conducted in three phases and, to limit the seasonal effect, surveys were conducted at the same time of the year in which the previous surveys had been done in each phase. Moreover, to understand the unintended consequences and other factors associated with good IYCF practices among the study population in a more holistic way, we analysed qualitative findings using the concurrent assessments, process evaluation and operations research conducted throughout the evaluation period. The concurrent qualitative assessment was conducted from October 2016 to January 2017; operations research was conducted from October 2015 to February 2016, and process evaluation was conducted from March 2016 to June 2017. Process evaluation and operations research were also conducted as part of the main evaluation of the MIYCN programme. For this study, we also used qualitative data from process evaluation and operations research. The evaluation method has been discussed in detail in other papers (Sarma, Uddin, Harbour, & Ahmed, 2016; Sarma et al., 2020a).

### 2.2 Programme description

BRAC, the largest NGO in Bangladesh, contributes to the improvement of IYCF practices through community health workers (CHWs) as an implementing partner of the IYCF programme of Alive and Thrive (A&T) in Bangladesh. Their large‐scale intervention, comprising a combination of interpersonal counselling, community mobilization, advocacy, mass communication and strategic use of data, has great potential to rapidly improve IYCF practices (Sanghvi et al., 2016). BRAC's Shasthya Shebika (SS)—female volunteer CHWs—makes door‐to‐door visits in their catchment areas, providing essential healthcare services, disseminating messages on maternal and child nutrition and selling various health products, such as paracetamol, iron‐folic acid, calcium, zinc tablets, oral rehydration salt (ORS), oral contraceptive pills, condoms, pregnancy strips, delivery kits, sanitary napkins and Pushtikona‐5 (a brand of micronutrient powder [MNP]). As part of BRAC's MIYCN programme (Sarma et al., 2020c) implemented from 2014 to 2018, BRAC's SS promoted IYCF practices as part of the home‐fortification of MNP interventions. To achieve high home‐fortification coverage, BRAC made several course corrections and programmatic changes during the implementation of this programme.

The course corrections and programmatic changes were as follows: (i) Four boxes of MNP were provided to CHWs free of charge as a source of resolving funds (resolving funds were among the income generation activities of CHWs); (ii) simple pictorial counselling cards were developed for CHWs for interactive counselling with caregivers; (iii) the incentive disbursement period was changed, and training on a microbusiness plan for CHWs was provided; (iv) refresher training strategies were revised; (v) performance‐based training for SKs (defined in the later section) was introduced; and (vi) the buffer stock of MNPs at the subdistrict level was ensured. In addition, in early 2016, BRAC removed SS's incentives for IYCF counselling. The incentives included BDT 50.0 (USD 0.59) for confirming the breastfeeding of a child within 1 h after birth, BDT 20.0 (USD 0.24) per month for confirming exclusive breastfeeding of a child for 6 months, BDT 10.0 (USD 0.12) for confirming the start of timely complementary feeding, BDT 5.0 (USD 0.059) for confirming the start of foods of animal source and BDT 5.0 for confirming hand washing. A detailed description of the programme and the course correction at different phases have been provided elsewhere (Sarma et al., 2020c).

### 2.3 Quantitative survey

#### 2.3.1 Sample size

We applied two sample‐size calculation formulas for the eight cross‐sectional surveys. Initially, during programme implementation periods, a total of five cross‐sectional surveys (three baseline and two midline surveys) were conducted to ensure the coverage of the programme. For these five surveys, we considered a 50% prevalence of MNP coverage, a precision of ±10% and a design effect of 2. Our estimated minimum sample size was 192 households per district for caregivers of 6‐ to 59‐month‐old children for the first five cross‐sectional surveys. At the end of the programme, three endline surveys were conducted at three different times (in alignment with the previous surveys) in the programme areas. Based on the baseline information regarding outcome indicators (including anaemia and good IYCF practices) obtained from the initial five surveys, we estimated sample size considering a 10% effect size of anaemia, 20% effect size of good IYCF practices, 90% power and a design effect of 2. We chose the prevalence of anaemia for the final sample‐size calculation since the sample size based on the prevalence of anaemia was the highest. For this article, we used data collected from caregivers of children aged 6 to 23 months who comprised 38.3% (*n* = 6,479) of the estimated total sample of 6‐ to 59‐month‐old children and their caregivers (*n* = 16,936).

#### 2.3.2 Sampling procedure

A two‐stage sampling procedure was applied for selecting study participants at the household level from the selected districts. In the first stage, systematic random sampling was used to select communities or primary sampling units (PSUs), with an equal selection probability for each community or PSU. Systematic samples of 16 PSUs were drawn from a complete list of the targeted communities or PSUs, which were sorted by district and by subdistrict within the programme districts (the programme districts for each phase were fixed and we conducted baseline, midline and endline surveys in the same districts of a phase) to reach the minimum estimated sample size. This ensured that all the target communities had an equal chance of being selected for the sample. In the second stage, a physical map‐segment sample approach was exercised to segment the selected communities or PSUs. At the final stage, the Expanded Programme on Immunization method was applied by spinning a bottle/pen placed in the centre of the segment, counting the households along that route and picking the fifth household. The selection of households depended on the eligibility criteria (caregivers of 6‐ to 23‐month‐old children, mothers/caregivers having resided there for at least 12 months before the day of the interview, the child not being physically challenged or ill). An Android‐based tablet (smartphone device) using the Open Data Kit software was used for collecting data.

#### 2.3.3 Outcome measures

The outcome variable in this study was IYCF practices (categorized as good IYCF practices and poor IYCF practices) among caregivers of 6‐ to 23‐month‐old children. Information on IYCF practices was obtained using a 24‐h recall questionnaire, and IYCF practices were assessed for children aged 6 to 23 months. A modified Infant and Child Feeding Index (ICFI) score of 6 was considered to indicate ‘good IYCF practices’, and any score less than 6 was rated as ‘poor IYCF practices’. The ICFI score was estimated based on age‐specific breastfeeding, age‐appropriate dietary diversity and age‐appropriate meal frequency (Guevarra et al., 2014). Details about the ICFI score calculation are presented in Table S1. We also performed a sensitivity analysis using different ICFI score cut‐offs (Table S2).

#### 2.3.4 Covariate measures

**FIGURE 1:**
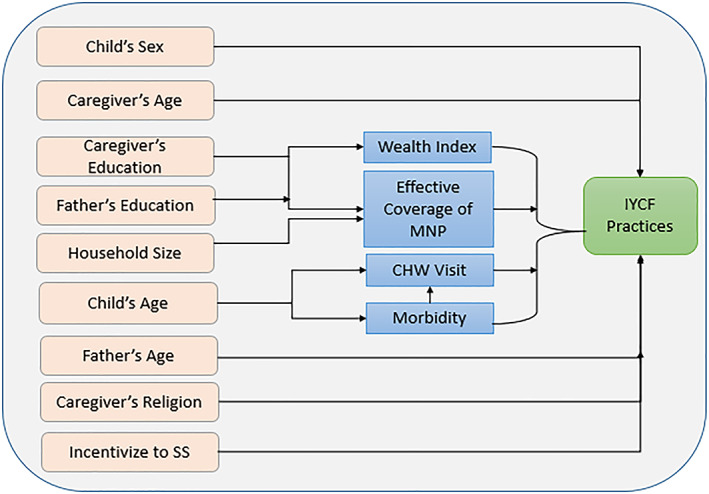
Conceptual framework of the hypothesized association of IYCF practices and other factors

The covariates were identified from the review of relevant literature (Agho et al., 2016; Epstein et al., 2019; Gautam et al., 2016; Issaka, Agho, Burns, Page, & Dibley, 2015; Kabir et al., 2012; Kassa et al., 2016; Khan et al., 2017; Khanal et al., 2013; Locks et al., 2017; Locks et al., 2018; Mihrshahi et al., 2010) and included the known ones that might have a potential effect on the outcome. The covariates for this study were household size (two categories: <5 and ≥5 members), child's sex (categorized as male and female), child's age (categorized as 6 to 11 months and 12 to 23 months), caregiver's age (categorized as <25 years and ≥25 years), caregiver's education (categorized as <5 years and ≥5 years), father's age (categorized as <30 years and ≥30 years), father's education (categorized as <5 years and ≥5 years), child morbidity status (any complications, such as diarrhoeal diseases, fever, difficulties or fast breathing with cough, breathing difficulties or fast breathing with blocked or running nose, breathing difficulties or fast breathing with cough and blocked or running nose in the last 14 days categorized as ‘yes’ and no such complications categorized as ‘no’), household visit from a CHW within the last 12 months (categorized as ‘yes’ and ‘no’), caregiver's religion (categorized as ‘Other’, including Hindu, Buddhist, Christian and ‘Muslim’), effective coverage of MNP (child fed three or more sachets of MNP in the 7 days before the day of interview: categorized as ‘yes’ if got effective coverage, otherwise ‘no’) and wealth index (categorized as poor, middle and rich). The wealth index was calculated by using household materials (e.g., materials used for the floor, roof and wall of the house) and household assets (including type of latrine used and sources of drinking water) by performing a principal component analysis (National Institute of Population Research and Training, ICF International,,, & Mitra and Associates, 2014). Additionally, we considered incentives for SSs (categorized as ‘yes’ if the SS received incentives for IYCF promotion and ‘no’ if the SS did not receive incentives). The household size, caregiver's age and father's age were categorized based on the median value (one category for values below the median and another for values at and above the median). We developed a conceptual framework to present the hypothesized association of good IYCF practices with other factors (Figure 1). Among the factors in the conceptual framework, child's sex, caregiver's age, caregiver's religion, father's age and incentivize to SS are directly linked to IYCF practices (Issaka et al., 2015; Kabir et al., 2012; Mukta & Ahmed, 2012). Before being linked to IYCF practices, wealth index and effective coverage of MNP depend on both father's and caregiver's education (Liu et al., 2018; Sarma et al., 2020a). Parental education has a potential role to improve wealth status and educated parents are more likely to feed MNP regularly to their children (Liu et al., 2018; Sarma et al., 2020a). The effective coverage of MNP is also linked to the household size (Sarma et al., 2020b). Household visit from a CHW and morbidity status of children depend on child's age because, usually, a CHW visits the households of younger children and the younger children are more likely to become ill compared to their older counterpart (Kamal, Hasan, & Davey, 2015; Sarma et al., 2020b). Thus, households visit from a CHW and morbidity status of the children are linked and further, both the CHW visit and morbidity status are linked with IYCF practices (Mukta & Ahmed, 2012; Sheikh et al., 2020).

#### 2.3.5 Data analysis

All statistical analyses were performed using the statistical software package Stata (Version 13). Quantitative data analyses included descriptions of the study population and estimates of different indicators related to IYCF practices and are presented in terms of frequency and percentage with 95% confidence intervals (CIs). We performed a chi‐square test to determine the differences in sociodemographic and background characteristics at the baseline, midline and endline surveys. We performed an adjusted Wald test to determine the differences in good IYCF practices at different time points of the survey. A simple generalized estimating equation (GEE) model was applied to determine the statistical associations between good IYCF practices and sociodemographic or other covariates by taking into account the adjustment of the clustering effect. The results are presented as risk ratios (RRs) with 95% CIs. Covariates that were significantly associated with good IYCF practices in the simple GEE model and also conceptually linked with good IYCF practices (even if they were not statistically significant in the simple GEE model) were included in the final multivariable GEE model. Finally, a multivariable GEE model was carried out to explore the factors associated with good IYCF practices. Cluster‐adjusted and weighted analyses were performed by using the ‘svy’ command. We checked the collinearity among the covariates and found that the variance inflation factor was below 1.50. A *p* value of <0.05 was considered as statistically significant.

### 2.4 Qualitative assessment

#### 2.4.1 Study participants and data collection

Qualitative data included in‐depth interviews (IDIs), focus group discussions (FGDs) with BRAC's SSs and their supervisors and key informant interviews (KIIs) with BRAC's programme personnel. We also performed IDIs with caregivers. Several qualitative studies were performed concurrently throughout the 5‐year evaluation study to understand the intended and unintended consequences of BRAC's MIYCN Phase 2 programme. To collect qualitative data, we purposively selected study participants from the BRAC MIYCN programme areas. We conducted IDIs with caregivers to understand their perceptions, knowledge and practices related to IYCF. IDIs and FGDs were also performed with BRAC's SSs and their supervisors; and KIIs were conducted with BRAC's programme personnel to understand their roles and responsibilities in the programme and the challenges they faced during service delivery. We also explored how they minimized and overcame those challenges during our investigation. The SSs, Shasthya Kormis (SKs), programme organizers (POs) and field organizers (FOs) were selected purposively, considering those who were involved in the MIYCN programme activities. The SKs were the supervisors of the SSs, and POs and FOs were the supervisors of both the SSs and SKs. The SSs and SKs were both BRAC community health workers, whereas the SKs, POs and FOs were the paid workers and the SSs were the voluntary workers of BRAC. In addition, BRAC's programme personnel, such as upazila managers (UMs) and district managers (DMs), were selected because they were involved in monitoring all the activities of their subordinates. A nine‐member team of investigators was involved in data‐collection processes; it included anthropologists and social scientists with academic and/or professional training. Interviews were audio‐recorded and transcribed immediately after the interview. A distinct anonymous identification code was assigned to each interview to maintain the confidentiality of the participant. We collected data from 13 districts, namely, Faridpur, Rajbari, Mymensingh, Manikganj, Rangpur, Kurigram, Gaibandha, Magura, Jashore, Chattogram, Cumilla, Feni and Sylhet across five divisions of Bangladesh (i.e., Dhaka, Rangpur, Khulna, Chattogram and Sylhet).

### 2.5 Data analysis

Data analysis was started at the very beginning of data collection and was performed in two phases: (i) during data collection and (ii) after data collection. All the data collectors and investigators were involved in the data analysis process. We used thematic analysis to triangulate and supplement the quantitative findings. To explore the influence on good IYCF practices through household visits and counselling, we analysed IDIs, FGDs with BRAC's SSs and their supervisors. Moreover, we analysed the KIIs with BRAC's programme personnel to gain a clear understanding of how IYCF activities were implemented under different programmes, how the programme priorities shifted over time and how these impacted the practices of CHWs and caregivers. All the qualitative interviews were transcribed and coded line by line prior to the thematic analysis.

### 2.6 Ethical considerations

The full study protocol (including survey questionnaire and consent form) was reviewed by the Institutional Review Board of icddr,b, which consisted of two committees: the Research Review Committee and the Ethical Review Committee. We obtained written informed consent from caregivers before conducting interviews. The interviewers read out the consent form to the respondents and answered any questions that arose, prior to obtaining written consent.

## 3 RESULTS

### 3.1 Sociodemographic and background characteristics

In this study, we included 2,633 child‐caregiver dyads at baseline, 1,762 at midline and 2,084 at endline. We selected children aged 6 to 23 months and their caregivers for our quantitative analyses. The sociodemographic and background characteristics of the caregivers, fathers and children aged 6 to 23 months were similar across the baseline, midline and endline surveys. We found that 78.0% of caregivers at baseline, 81.1% of caregivers at midline and 82.3% of caregivers at endline survey had completed 5 or more years of schooling. Approximately 62% of children at baseline, 64.9% of children at midline and 54.8% of children at endline experienced morbidity within the 14 days before the day of the interview (Table 1). For the qualitative assessment, we conducted 27 IDIs, 10 FGDs with BRAC CHWs and 18 KIIs with BRAC programme personnel. We also performed 38 IDIs with caregivers.

**TABLE 1:** Sociodemographic and background characteristics of the study participants^a^

Characteristics	Baseline (*n* = 2,633)	Midline (*n* = 1762)	Endline (*n* = 2084)	*p* value^b^
	*n* (%)	*n* (%)	*n* (%)	
Household characteristics				
Household‐size				
≥5 members	1,584 (60.1)	1,075 (61.3)	1,266 (60.1)	0.752
Wealth index				
Poor	873 (33.9)	622 (35.1)	719 (33.4)	0.890
Middle	884 (32.0)	549 (31.1)	673 (32.9)	
Rich	876 (34.2)	591 (33.8)	692 (33.7)	
Caregiver's characteristics				
Caregiver's age				
≥25 years	1,373 (52.1)	943 (52.8)	1,142 (54.8)	0.300
Caregiver's education				
≥5 years	2,036 (78.0)	1,395 (80.1)	1,711 (82.3)	0.021
Caregiver's religion				
Muslim	2,374 (90.3)	1,551 (88.1)	1,873 (90.3)	0.346
Father's characteristics				
Father's age				
≥30 years	1,687 (64.9)	1,182 (66.6)	1,429 (68.5)	0.109
Father's education				
≥5 years	1703 (65.6)	1,175 (68.4)	1,411 (67.4)	0.213
Child's characteristics				
Child's age				
12 to 23 months	1,763 (67.3)	1,179 (66.6)	1,460 (70.3)	0.107
Child's sex				
Male	1,394 (53.8)	911 (51.6)	1,052 (50.6)	0.123
Child's morbidity status				
Yes	1,637 (62.4)	1,121 (64.9)	1,162 (54.8)	<0.001

^a^ Analyses were weighted based on Villages (PSUs) population size.

^b^ *p* values were generated from chi‐square test.

### 3.2 Inconsistent trend in the prevalence of good IYCF practices

**FIGURE 2:**
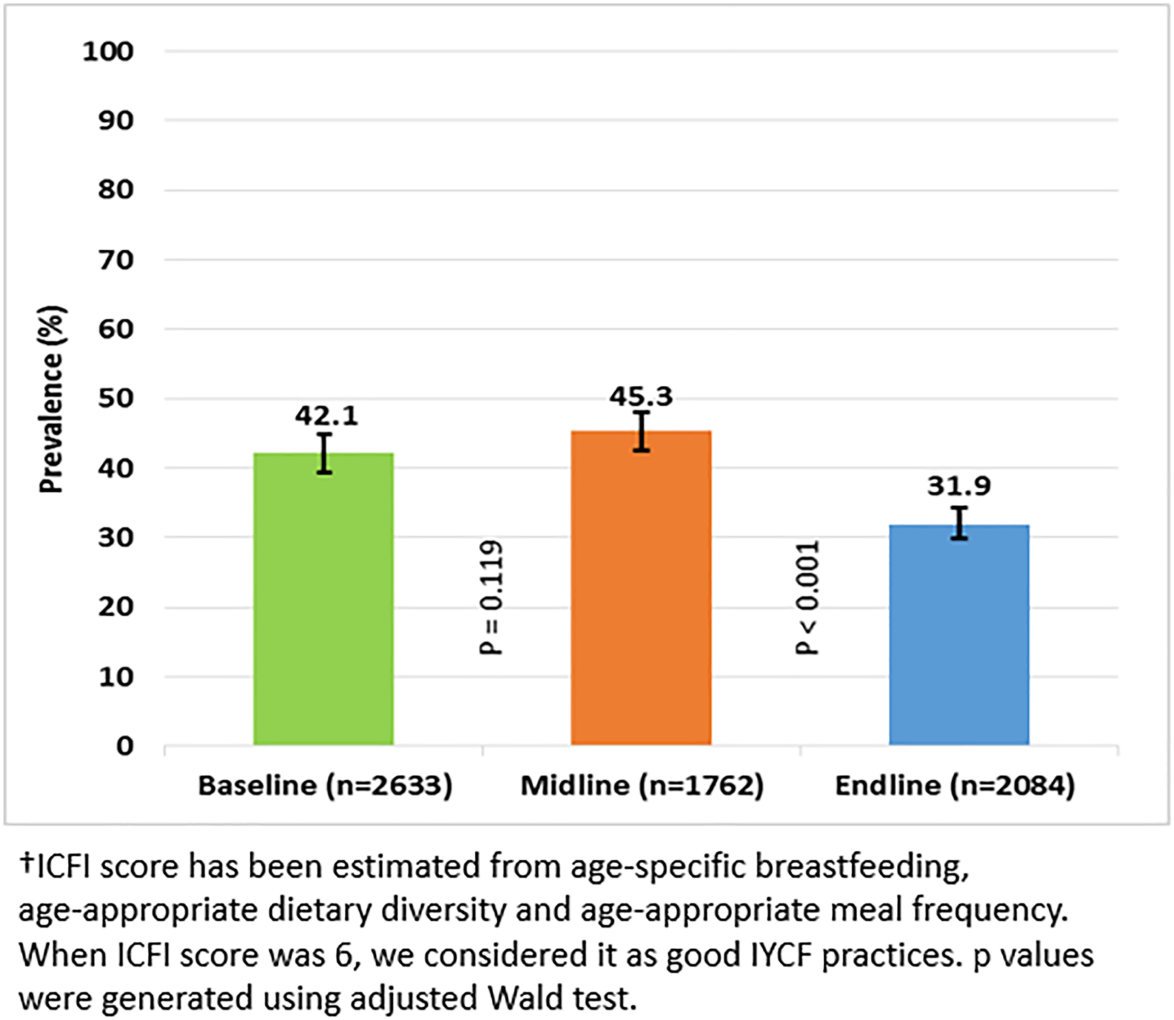
Prevalence of good infant and young child feeding (IYCF) practices based on ICFI^†^

**FIGURE 3:**
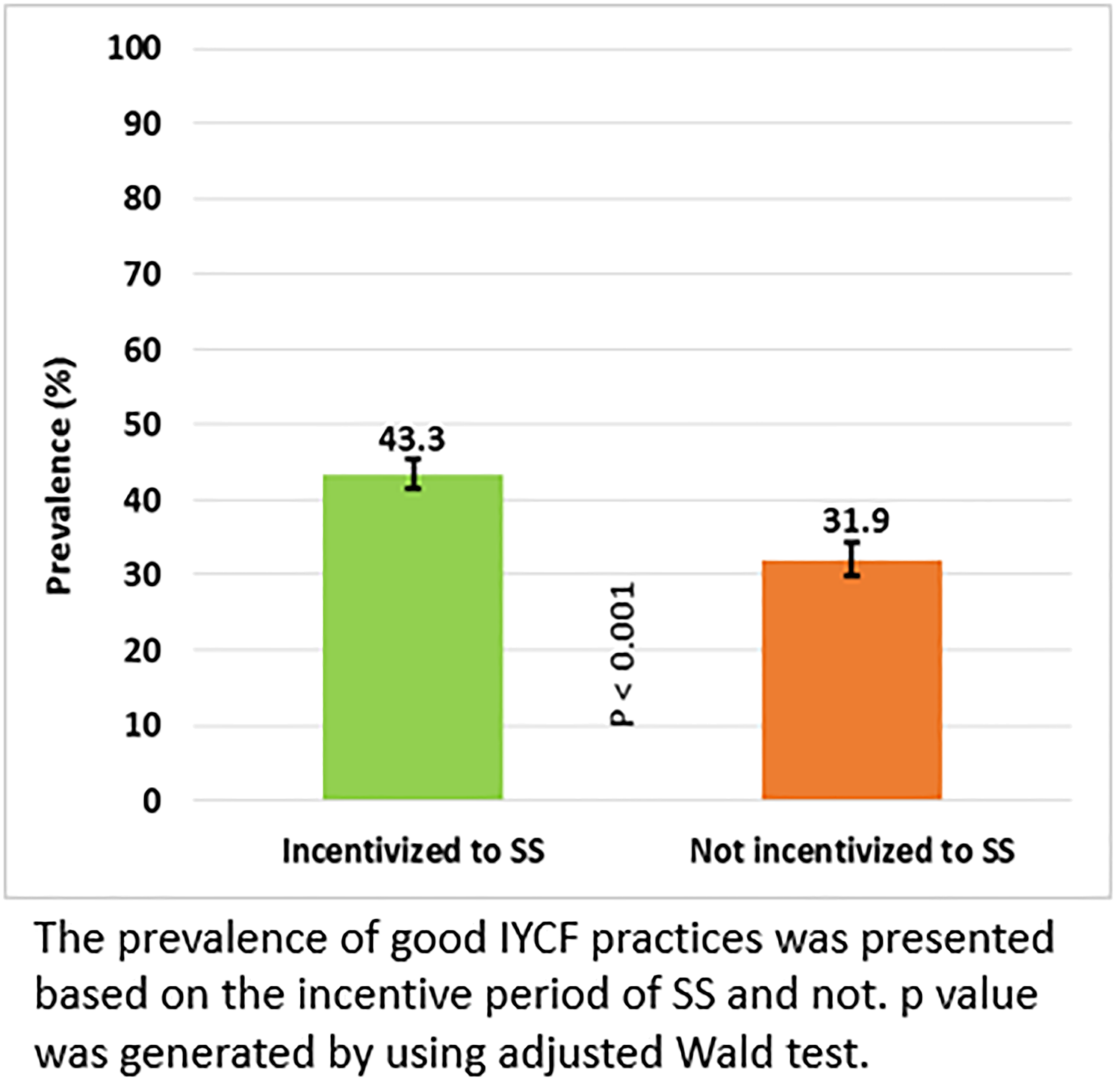
Prevalence of good infant and young child feeding (IYCF) practices when SSs received incentives for IYCF promotion and when they did not receive incentives for IYCF promotion

**FIGURE 4:**
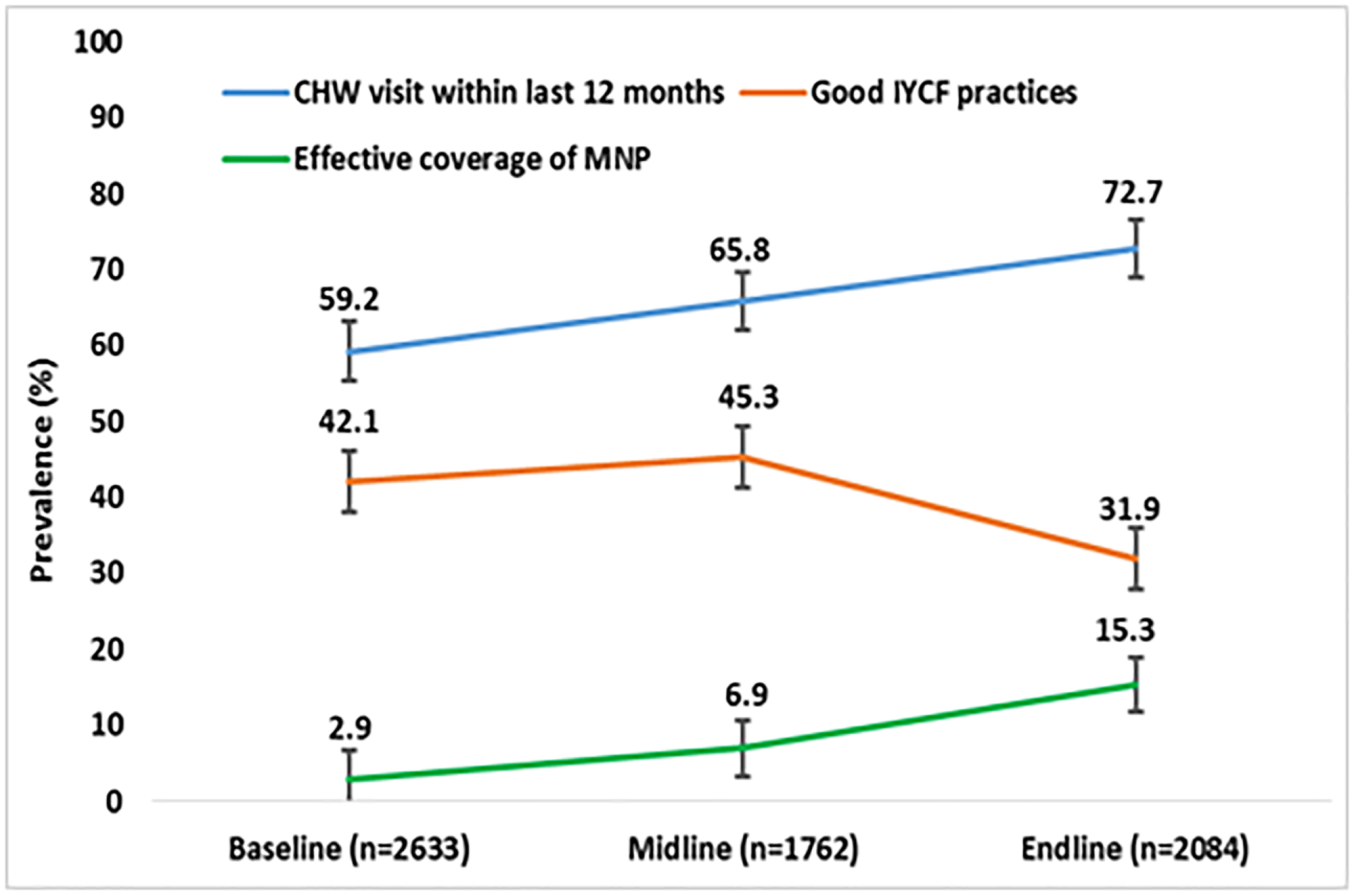
Prevalence of programmatic indicators at baseline, midline and endline

Although the programme was targeted to promote IYCF, the prevalence of good IYCF practices was 42.1% at baseline and increased to 45.3% at midline but decreased significantly (*p* < 0.001) to 31.9% at endline (Figure 2). Figure 3 illustrates the prevalence of good IYCF practices before and after the cessation of incentives for promoting IYCF. The incentives for the BRAC SSs were specifically aimed at promoting IYCF and were removed in early 2016, and we found that before they ended, the prevalence of good IYCF practices was 43.3%, it decreased to 31.9% (*p* < 0.001) when incentives to SSs for IYCF promotion ended. Figure 4 displays the prevalence of CHW visits, good IYCF practices and effective coverage of MNP at different time points. The rate of CHW visits was 59.2% at baseline, 65.8% at midline and 72.7% at endline survey. Only approximately 3% of caregivers at baseline, 7% at midline and 15% at endline fed their children three or more sachets of MNP (effective coverage) in the 7 days prior to the interview.

Our qualitative findings (end of 2015) revealed how CHW explained and demonstrated the process of feeding complementary foods with MNP. The CHW advised the mothers to give any family food like fish, meat or eggs by smashing those like paste to make the taste familiar to the baby, in addition to advice on to feed food mixed with MNP. One packet of MNP has to be given with one meal at a time. The CHW read them out loudly the directions written in the packet; initially, CHW demonstrates how to prepare it by mixing the MNP. Our concurrent qualitative investigations (end of 2016) explored that mothers were knowledgeable about feeding their children with complementary foods from the age of 6 months besides breastfeeding. A mother stated, ‘I have been giving breast milk as well as extra food through Khichuri (a dish made of rice and lentils) to both of my children from their 6 months of age. I used to cook the rice, lentils mixed with green vegetables, pumpkins, carrots, eggs, and fed my sons’.

Although CHW visit and effective coverage of MNP increased from baseline to midline and endline, the good IYCF practices decreased from baseline and midline to endline because the programme changed its focus from promoting IYCF to promoting MNP use. Therefore, CHWs increased their visit frequency but concentrated on MNP promotion instead of IYCF promotion. The phasewise prevalence of good IYCF practices is presented in Figure S2. We also present the prevalence of the components of IYCF indicators including continued breastfeeding, age‐appropriate dietary diversity and age‐appropriate meal frequency in Figure S3.

### 3.3 Factors associated with good IYCF practices

Table 2 presents the factors associated with good IYCF practices among caregivers of 6‐ to 23‐month‐old children. The results of the multivariable GEE in model 4 revealed that during the period when SSs were not incentivized to promote IYCF, the probability of maintaining good IYCF practices among caregivers was 28% (ARR: 0.72; 95% CI [0.67, 0.78]) lower than during the period when SSs were incentivized. Model 4 revealed that caregivers in households that were visited by BRAC CHWs were 13% (ARR: 1.13, 95% CI [1.05, 1.21]) more likely to adopt good IYCF practices than households that were not visited by CHWs. However, we found a significant association between good IYCF practices and CHW visits in Model 1 (Baseline) and Model 2 (Midline), and this association was not significant in Model 3 (Endline), indicating that visits had no significant effect on IYCF at endline because, during this period, SSs were not incentivized to promote IYCF. Caregivers of children who received effective coverage of MNP were 21% (ARR: 1.21; 95% CI [1.10, 1.33]) more likely to adopt good IYCF practices than caregivers of children who did not receive effective coverage of MNP; however, caregivers of morbid children were 10% (ARR: 0.90; 95% CI [0.84, 0.95]) less likely to adopt good IYCF practices.

**TABLE 2:** Multivariable GEE model of associated factors of good IYCF practices among caregivers of 6 to 23‐month‐old children

Variable	Baseline (Model 1)	Midline (Model 2)	Endline (Model 3)	Pooled data (Model 4)
	ARR (95% CI)	ARR (95% CI)	ARR (95% CI)	ARR^a^ (95% CI)
Household size				
<5 members	—	Ref.	Ref.	Ref.
≥5 members	—	1.02 (0.92, 1.14)	1.08 (0.95, 1.23)	1.05 (0.98, 1.11)
Child's sex				
Male	—	Ref.	Ref.	Ref.
Female	—	0.93 (0.85, 1.02)	0.93 (0.83, 1.04)	0.94^*^ (0.89, 0.99)
Child's age				
6 to 11 months	Ref.	Ref.	Ref.	Ref.
12 to 23 months	0.61^***^ (0.56, 0.67)	0.69^***^ (0.62, 0.77)	0.40^***^ (0.35, 0.46)	0.57^***^ (0.53, 0.60)
Caregiver's religion				
Hindu and others	Ref.	Ref.	Ref.	Ref.
Muslim	0.77^***^ (0.68, 0.88)	0.83^*^ (0.72, 0.96)	0.75^**^ (0.63, 0.90)	0.79^***^ (0.73, 0.87)
Caregiver's age				
<25 years	—	Ref.	Ref.	Ref.
≥25 years	—	0.96 (0.86, 1.06)	0.92 (0.82, 1.04)	0.99 (0.93, 1.05)
Caregiver's education				
<5 years	Ref.	Ref.	Ref.	Ref.
≥5 years	1.15^*^ (1.01, 1.31)	1.02 (0.89, 1.18)	1.09 (0.91, 1.29)	1.10^*^ (1.01, 1.19)
Father's education				
<5 years	Ref.	Ref.	Ref.	Ref.
≥5 years	1.24^***^ (1.11, 1.38)	1.15^*^ (1.01, 1.30)	1.20^*^ (1.02, 1.40)	1.19^***^ (1.10, 1.28)
Wealth index				
Poor	Ref.	Ref.	Ref.	Ref.
Middle	0.96 (0.86, 1.07)	1.15^*^ (1.00, 1.31)	1.06 (0.91, 1.23)	1.03 (0.96, 1.11)
Rich	1.02 (0.92, 1.14)	1.25^**^ (1.10, 1.43)	1.19^*^ (1.02, 1.39)	1.13^**^ (1.05, 1.22)
Morbidity status				
No	Ref.	Ref.	Ref.	Ref.
Yes	0.87^**^ (0.80, 0.95)	0.88^*^ (0.79, 0.97)	0.93 (0.83, 1.05)	0.90^***^ (0.84, 0.95)
CHW's visit within the last 12 months				
No	Ref.	Ref.	Ref.	Ref.
Yes	1.15^**^ (1.04, 1.27)	1.16^*^ (1.03, 1.30)	1.10 (0.95, 1.27)	1.13^***^ (1.06, 1.21)
Effective coverage of MNP				
No	Ref.	Ref.	Ref.	Ref.
Yes	1.11 (0.91, 1.35)	1.15 (0.99, 1.34)	1.30^**^ (1.12, 1.51)	1.21^***^ (1.10, 1.33)
Incentivized to SS for IYCF promotion				
Yes	—	—	—	Ref.
No	—	—	—	0.72^***^ (0.67, 0.78)

^*^ *p* < 0.05.

^**^ *p* < 0.01.

^***^ *p* < 0.001.

^a^ Adjusted risk ratios, 95% confidence intervals and *p* values were obtained from log‐binomial regression model using generalized estimating equation (simple GEE model [Table S3]). When the log‐binomial model would not converge, a log‐Poisson model was used (multivariable GEE model). At baseline and endline surveys, respectively, effective coverage and CHW's visit within 12 months were insignificant in simple GEE model, but we included in the multivariable GEE model because these were confounders.

Our qualitative findings also revealed that some unintended consequences (an inconsistent trend in the prevalence of good IYCF practices) may have stemmed from programmatic changes (i.e., variations in the provision of incentives at different timepoints and for different programme components). Qualitative assessment conducted at the midpoint (2016) of the MIYCN programme revealed that some programmatic factors discouraged the SSs from performing IYCF‐related activities. A BRAC programme organizer described this effect:

During the Alive and Thrive IYCF programme, if any SS could confirm exclusive breastfeeding of a child for six months, she received BDT 20.0 per month. Now, they are working under the nutrition programme and doing the same service. However, their incentives have been removed. It demotivated them compared to the previous time.

Qualitative analysis also revealed that CHWs emphasized the sale of Pushtikona‐5 over counselling caregivers about IYCF because they received incentives for the sale of Pushtikona‐5. One of the CHWs stated the following:

I earn more by selling Pushtikona‐5; for this reason, I emphasize more on selling of this compared to other health products, and I try to counsel and motivate the caregivers by focusing on the benefits of Pushtikona‐5.

Model 4 also revealed that caregivers who had 12‐ to 23‐month‐old children were 43% (ARR: 0.57; 95% [CI 0.53, 0.60]) less likely to maintain good IYCF practices than caregivers of 6‐ to 11‐month‐old children. Caregivers who were Muslim were 21% (ARR: 0.79; 95% CI [0.73, 0.87]) less likely to maintain good IYCF practices than caregivers of other religions. Although the caregivers in Muslim families adopted fewer good IYCF practices than families of Hindus or another religion, the qualitative investigation revealed that the Hindu communities generally offered the first meal to girls and boys respectively at 7 and 8 months of age in accordance with a Hindu ritual called Onnoprashon/Annaprashana, which marks an infant's first intake of food other than milk. This is evidence of a barrier of good IYCF practices, which is compromised by this age‐specific dietary diversity indicator. We further found from Model 4 that fathers and caregivers with five or more years of schooling were more likely to adopt good IYCF practices (ARR: 1.19 for father and ARR: 1.10 for caregivers). We also present the factors associated with the IYCF indicators including, continued breastfeeding, age‐appropriate dietary diversity and age‐appropriate meal frequency in Table S4.

Apart from these factors (child's sex, child's age, caregiver's religion, caregiver's education, father's education, wealth index, morbidity status, CHW visits within the last 12 months and effective coverage of MNP) associated with good IYCF practices, the qualitative findings revealed that the caregiver's attitude influenced complementary feeding practices; economic constraints and lack of appetite were often more important factors in these practices than age‐appropriate dietary diversity or age‐appropriate meal frequency. Caregivers considered giving foods other than breast milk to their children after a certain age; this practice varied from one child to another. Qualitative findings also revealed that older members (e.g., grandmothers) of the family usually believed complementary foods should be started when mothers were unable to produce sufficient breast milk. The caregivers often did not take into account which foods provided proper nutrition or which foods were appropriate for a minimum acceptable diet, and many of them had little knowledge of what age‐appropriate dietary diversity meant. They believed that the children should be offered food that sated their hunger or according to their taste. The findings also revealed how caregiver's perception influenced good IYCF practices, despite the presence of an intervention in a particular programme area. A caregiver (mother) of a 14‐month‐old child said:

I feed my child according to his taste. Now I give hotchpotch, as he likes it. That practice continued for 2‐4 months. I fed him rice powder until he was aged 8‐9 months. I have been feeding him hotchpotch and, sometimes, rice since the age of nine months.

## 4 DISCUSSION

Household data from different representative parts of Bangladesh where the MIYCN programme of BRAC was implemented were analysed in this study to understand the unintended consequences of programmatic changes in IYCF practices. We found that the prevalence of good IYCF practices at the endline survey was approximately 32%, whereas the nationally representative data for Bangladesh (BDHS 2014) showed that 23% of caregivers maintained IYCF practices, which is still suboptimal. The results of this study indicate a significant decrease in good IYCF practices from baseline–midline to endline. Unintended consequences of programmatic changes were the major factors for this decreasing pattern. The reason might stem from the sudden decrease in incentives for CHWs after the withdrawal of funding under the A&T programme, which was specifically intended to improve IYCF practices. The A&T programme was the intervention programme implemented in Bangladesh from 2009 to 2014 to improve IYCF practices among the caregivers of children under 5 years of age (Menon et al., 2016; Sanghvi et al., 2016). Performance‐based monetary incentives awarded to SSs changed their performance on IYCF messaging and improved IYCF practices (Mukta & Ahmed, 2012). Furthermore, most of the interventions were designed considering the sale and promotion of MNP rather than the improvement of IYCF practices. However, greater emphasis on increasing MNP coverage by SSs may result in a lack of attention to IYCF counselling.

Our pooled data findings show that CHW visits within the last 12 months were a potential positive confounder of good IYCF practices among caregivers. We also found a significant association of CHW visits with good IYCF practices at baseline and midline but a nonsignificant association with the results of the endline survey. This might be because the baseline and midline surveys were carried out when CHWs were incentivized to promote IYCF, but the endline survey was conducted during the period when the incentive was removed. This demotivated the CHWs to provide counselling on and demonstrations of IYCF practices; as a result, optimal IYCF practices were decreased at the endline survey even though the number of CHW visits increased. A study conducted in Bangladesh showed that IYCF counselling by frontline health workers had a significant effect on improving exclusive breastfeeding rates and minimum dietary diversity (Epstein et al., 2019). Another study, in Madagascar, showed that mothers who attended a meeting at which a CHW talked about IYCF were one and half times as likely to feed MNP to their children (Locks et al., 2017), which partially supports the role of CHWs in improving IYCF practices. However, a sudden decrease in incentives for the CHWs after the withdrawal of funding during the A&T Phase 1 programme had a potential influence on CHW visits for IYCF counselling in the programme areas, a practice that was specifically intended to improve IYCF practices.

In the MIYCN programme of BRAC, the SSs emphasized the sale of MNP more than counselling on IYCF because they received incentives for selling MNP, and not for providing counselling on IYCF. It might be that the CHWs prioritized MNP promotion over IYCF promotion based on incentives. It has been reported that when incentives are provided for a particular practice, they can lead to low performance in routine health services among health workers (Griffiths et al., 2011). An intervention that integrates MNP promotion with an increased number of household visits could play a significant role in improving IYCF practices (Locks et al., 2017).

We found that caregivers and fathers with 5 or more years of schooling had a higher probability of adopting good IYCF practices than did those who were illiterate or had less than 5 years of schooling. Similar evidence was found in a study conducted in Nepal (Gautam et al., 2016). Some other studies have revealed that the low level of maternal knowledge in South Asia is associated with inappropriate IYCF practices (Senarath, Agho, et al., 2012; Victor, Baines, Agho, & Dibley, 2014). This may be why educated parents have both adequate knowledge of and greater awareness regarding feeding practices. Our qualitative findings indicate that caregiver's perceptions of complementary feeding practices could also vary among different areas and could thus contribute to inconsistent patterns of IYCF practices across programme areas. A study recently conducted in Bangladesh illustrates that complementary feeding practices are related to mothers' perceptions (Naila et al., 2018).

Poverty is a curse for all development processes, including the early physical, mental and cognitive development of children (Engle & Black, 2008; Park, Fertig, & Allison, 2011). The association between poor IYCF practices and the wealth index is well‐established (Khan et al., 2017). In this study, we also found that in terms of wealth index, a rich group of caregivers was more likely to adopt good IYCF practices compared with the poor caregivers. This finding has also been reported in other studies across different countries (Kabir et al., 2012; Senarath, Godakandage, et al., 2012; Victor et al., 2014; Zongrone, Winskell, & Menon, 2012).

This study found that caregivers practicing Hinduism and other religions paid greater attention to their children's feeding practices than did those who were Muslims. We did not find any literature in the Bangladesh context that corroborates this difference. However, there was evidence in Ghana that Muslim and non‐Christian mothers had a higher risk of not introducing complementary foods than Christian mothers did (Issaka et al., 2015). Caregivers who had given at least three MNP sachets (effective coverage of MNP) to their children in the week prior to the interview day had a greater likelihood of adopting good IYCF practices. This could be because caregivers who regularly gave MNP to their children were more aware of IYCF practices. Some studies have found that caregivers who fed MNP to their children had improved IYCF practices for their children (Locks et al., 2018; Mirkovic et al., 2016; Siekmans et al., 2017), which supports our findings.

This study has some limitations. First, the selected sample size was a subset of the total estimated sample‐size, which reduced our estimation power. Second, we assessed IYCF practices based on recall data, which may lead to recall bias. Last, this study was carried out in only some areas of Bangladesh; therefore, the findings should not be generalized to the whole country. Our qualitative findings also have some limitations. Some of the thematic findings were generated from a single data collection method, and we were unable to triangulate the results using data from other methods. Each of the studies had its own research objective; therefore, extracting IYCF practice‐related data was challenging when home‐fortification with MNP was the main area of focus. Moreover, some data were collected years after the caregivers actually engaged in these IYCF practices, which is likely to add some respondent bias. In addition, we were unable to explore the service providers experience with the day‐to‐day challenges of ensuring that caregivers engaged in good IYCF practices; therefore, we missed the opportunity for potential triangulation with their insights.

## 5 CONCLUSION

This study reported that the prevalence of good IYCF practices among caregivers decreased from both baseline and midline to the endline survey due to the unintended consequences of the programmatic changes. When designing and implementing a programme, careful measures should be taken to avoid unintended negative consequences, to ensure the effective implementation of the programme. Aside from the unintended consequences, the positive association of effective coverage of MNP with good IYCF practices suggests that the integration of MNP into IYCF interventions can achieve an optimal level of good IYCF practices. Moreover, attention should be paid to providing household visits by CHWs when designing such an integrated programme.

## CONFLICTS OF INTEREST

All authors declared that they have no conflict of interest.

## AUTHOR CONTRIBUTIONS

MT and HS conceptualized the paper. HS is the principal investigator of the evaluation and supervised MT for developing this manuscript. MT and GK analysed the quantitative data, SKL and MR analysed and interpreted the qualitative results, MT prepared the first draft and TA, HS, MR, SKL and GK critically reviewed and commented on the subsequent and final version of the manuscript.

## Supporting information


**Figure S1:** Timeline of different surveys at different phases
Figure S2 Prevalence of good IYCF practices at baseline, midline and endline in all phases
Figure S3 Prevalence of the components of IYCF indicators at baseline, midline and endline survey
Table S1: Variables and scoring systems used in constructing the Infant and Child Feeding Index
Table S2 Prevalence of good IYCF practices using different cut‐offs of ICFI score (sensitivity analysis)
Table S3 Simple GEE model of associated factors of good IYCF practices among caregivers of 6–23 months old children
Table S4 Multivariable GEE model of associated of IYCF indicators (using pooled data)
Table S5 Multiple GEE model of associated factors of good IYCF practices among caregivers of 6–23 months old children based on incentive to SS and not
Table S6 General Guideline for Qualitative Data Collection (Service Providers)
Table S7 General Guideline for Qualitative Data Collection (Caregivers)
