# Supplementary material for: Unintended consequences of programmatic changes to infant and young child feeding practices in Bangladesh
Source: Matern Child Nutr. 2020 Oct 16;17(2):e13077. doi: 10.1111/mcn.13077 (PMC7988846; doi:10.1111/mcn.13077)
Supplement: Supplementary file 1 — Figure S1: Timeline of different surveys at different phases Figure S2 Prevalence of good IYCF practices at baseline, midline and endline in all phases Figure S3 Prevalence of the components of IYCF indicators at baseline, midline and endline survey Table S1: Variables and scoring systems used in constructing the Infant and Child Feeding Index Table S2 Prevalence of good IYCF practices using different cut‐offs of ICFI score (sensitivity analysis) Table S3 Simple GEE model of associated factors of good IYCF practices among caregivers of 6–23 months old children Table S4 Multivariable GEE model of associated of IYCF indicators (using pooled data) Table S5 Multiple GEE model of associated factors of good IYCF practices among caregivers of 6–23 months old children based on incentive to SS and not Table S6 General Guideline for Qualitative Data Collection (Service Providers) Table S7 General Guideline for Qualitative Data Collection (Caregivers) [file MCN-17-e13077-s001.docx]

**Figure S1:** Timeline of different surveys at different phases


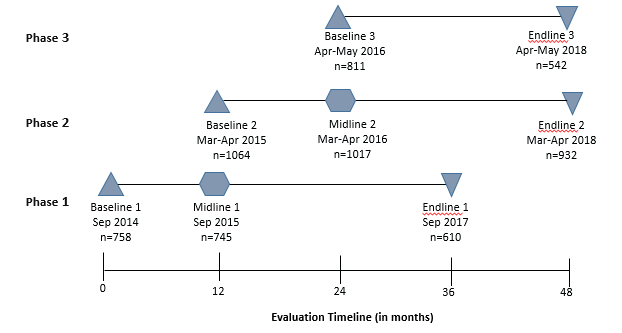


**Figure S2** Prevalence of good IYCF practices at baseline, midline and endline in all phases

**Figure S3** Prevalence of the components of IYCF indicators at baseline, midline and endline survey

**Table S1:** Variables and scoring systems used in constructing the Infant and Child Feeding Index

| **Indicators** | **Age groups (months)** | | | | | |
| --- | --- | --- | --- | --- | --- | --- |
|  | **6-8** | | **9-11** | | **12-23** | |
|  | **Value** | **Score** | **Value** | **Score** | **Value** | **Score** |
| Currently breastfed | yes | +2 | yes | +2 | yes | +1 |
| Food groups | 1  ≥2 | +1  +2 | 1 or 2  ≥3 | +1  +2 | 2 or 3  ≥4 | +1  +2 |
| Meal frequency | 1  ≥2 | +1  +2 | 1 or 2  ≥3 | +1  +2 | 2  3  ≥4 | +1  +2  +3 |

**Table S2** Prevalence of good IYCF practices using different cut-offs of ICFI score (sensitivity analysis)

| **ICFI score**  **Different cut-offs** | **Baseline, % (n)**  **N=2633** | **Midline, % (n)**  **N=1762** | **Endline, % (n)**  **N=2084** |
| --- | --- | --- | --- |
| >=3 | 95.3 (2513) | 96.0 (1690) | 96.4 (1998) |
| >=4 | 89.0 (2351) | 90.7 (1600) | 89.3 (1853) |
| >=5 | 71.7 (1896) | 76.6 (1358) | 67.8 (1407) |

**Table S3** Simple GEE model of associated factors of good IYCF practices among caregivers of 6-23 months old children

| **Variable** | **Baseline** | **Midline** | **Endline** |
| --- | --- | --- | --- |
|  | **RR (95% CI)** | **RR (95% CI)** | **RR (95% CI)** |
| **Household-size** |  |  |  |
| <5 members | Ref. | Ref. | Ref. |
| ≥5 members | 1.06 (0.96, 1.17) | 1.07 (0.96, 1.18) | 1.15* (1.01, 1.31) |
| **Child’s sex** |  |  |  |
| Male | Ref. | Ref. | Ref. |
| Female | 0.95 (0.87, 1.04) | 0.91 (0.83, 1.00) | 0.94 (0.84, 1.07) |
| **Child’s age** |  |  |  |
| 6- to 11 months | Ref. | Ref. | Ref. |
| 12- to 23 months | 0.60*** (0.54, 0.65) | 0.68*** (0.61, 0.75) | 0.40*** (0.35, 0.46) |
| **Caregiver’s religion** |  |  |  |
| Hindu and others | Ref. | Ref. | Ref. |
| Muslim | 0.75*** (0.66, 0.85) | 0.83* (0.72, 0.96) | 0.77** (0.64, 0.92) |
| **Caregiver’s age** |  |  |  |
| <25 years | Ref. | Ref. | Ref. |
| ≥25 years | 0.97 (0.89, 1.06) | 0.90* (0.81, 0.99) | 0.90 (0.80, 1.02) |
| **Caregiver’s education** |  |  |  |
| <5 years | Ref. | Ref. | Ref. |
| ≥5 years | 1.30*** (1.15, 1.48) | 1.19** (1.05, 1.36) | 1.24* (1.04, 1.48) |
| **Father’s age** |  |  |  |
| <30 years | Ref. | Ref. | Ref. |
| ≥30 years | 1.00 (0.91, 1.10) | 0.96 (0.86, 1.06) | 0.93 (0.82, 1.06) |
| **Father’s education** |  |  |  |
| <5 years | Ref. | Ref. | Ref. |
| ≥5 years | 1.29*** (1.17, 1.43) | 1.27*** (1.14, 1.42) | 1.33*** (1.14, 1.55) |
| **Wealth index** |  |  |  |
| Poor | Ref. | Ref. | Ref. |
| Middle | 1.00 (0.89, 1.12) | 1.20** (1.05, 1.37) | 1.08 (0.92, 1.27) |
| Rich | 1.13* (1.01, 1.27) | 1.35*** (1.18, 1.53) | 1.26** (1.08, 1.47) |
| **Morbidity status** |  |  |  |
| No | Ref. | Ref. | Ref. |
| Yes | 0.87** (0.80, 0.96) | 0.89* (0.80, 0.99) | 0.98 (0.86, 1.11) |
| **CHW’s visit within the last 12 months** |  |  |  |
| No | Ref. | Ref. | Ref. |
| Yes | 1.28*** (1.16, 1.42) | 1.20** (1.07, 1.36) | 1.07 (0.93, 1.24) |
| **Effective coverage of MNP** |  |  |  |
| No | Ref. | Ref. | Ref. |
| Yes | 1.19 (0.96, 1.48) | 1.28** (1.10, 1.50) | 1.37*** (1.18, 1.59) |

*p <0.05, ** p<0.01, ***p<0.001

**Table S4** Multivariable GEE model of associated of IYCF indicators (using pooled data)

| **Variable** | **Continued breastfeeding** | **Age-appropriate dietary diversity** | **Age-appropriate meal frequency** |
| --- | --- | --- | --- |
|  | **RR (95% CI)** | **RR (95% CI)** | **RR (95% CI)** |
| **Household-size** |  |  |  |
| <5 members | - | Ref. | - |
| ≥5 members | - | 1.02 (0.99, 1.06) | - |
| **Child’s sex** |  |  |  |
| Male | - | Ref. | - |
| Female | - | 0.96* (0.93, 0.99) | - |
| **Child’s age** |  |  |  |
| 6- to 11 months | Ref. | Ref. | Ref. |
| 12- to 23 months | 0.94*** (0.94, 0.96) | 0.89*** (0.86, 0.92) | 0.70*** (0.67, 0.73) |
| **Caregiver’s religion** |  |  |  |
| Hindu and others | Ref. | Ref. | Ref. |
| Muslim | 0.96*** (0.94, 0.97) | 0.90*** (0.85, 0.95) | 0.92** (0.86, 0.97) |
| **Caregiver’s age** |  |  |  |
| <25 years | - | - | - |
| ≥25 years | - | - | - |
| **Caregiver’s education** |  |  |  |
| <5 years | - | Ref. | - |
| ≥5 years | - | 1.08** (1.02, 1.14) | - |
| **Father’s age** |  |  |  |
| <30 years | - | - | - |
| ≥30 years | - | - | - |
| **Father’s education** |  |  |  |
| <5 years | - | Ref. | - |
| ≥5 years | - | 1.15*** (1.10, 1.20) | - |
| **Wealth index** |  |  |  |
| Poor | Ref. | Ref. | Ref. |
| Middle | 1.00 (0.98, 1.01) | 1.08** (1.03, 1.13) | 1.03 (0.98, 1.08) |
| Rich | 0.96*** (0.94, 0.98) | 1.17*** (1.11, 1.22) | 1.12*** (1.06, 1.17) |
| **Morbidity status** |  |  |  |
| No | - | Ref. | - |
| Yes | - | 0.91*** (0.88, 0.94) | - |
| **CHW’s visit within the last 12 months** |  |  |  |
| No | Ref. | Ref. | Ref. |
| Yes | 1.04*** (1.02, 1.05) | 1.07** (1.03, 1.12) | 0.99 (0.95, 1.03) |
| **Effective coverage of MNP** |  |  |  |
| No | Ref. | Ref. | Ref. |
| Yes | 1.001 (0.98, 1.02) | 1.16** (1.10, 1.22) | 1.12*** (1.04, 1.20) |
| **Incentivized to SS for IYCF promotion** |  |  |  |
| Yes | Ref. | Ref. | Ref. |
| No | 1.01 (0.99, 1.03) | 0.98 (0.94, 1.02) | 0.71*** (0.67, 0.76) |

*p <0.05, ** p<0.01, ***p<0.001

**Table S5** Multiple GEE model of associated factors of good IYCF practices among caregivers of 6-23 months old children based on incentive to SS and not

| **Variable** | **Incentivized to SS** | **Not Incentivized to SS** |
| --- | --- | --- |
|  | **ARR (95% CI)** | **ARR (95% CI)** |
| **Household-size** |  |  |
| <5 members | Ref. | Ref. |
| ≥5 members | 1.04 (0.97, 1.11) | 1.08 (0.95, 1.23) |
| **Child’s sex** |  |  |
| Male | Ref. | Ref. |
| Female | 0.95 (0.89, 1.01) | 0.93 (0.83, 1.04) |
| **Child’s age** |  |  |
| 6- to 11 months | Ref. | Ref. |
| 12- to 23 months | 0.64*** (0.60, 0.69) | 0.40*** (0.35, 0.46) |
| Caregiver’s religion |  |  |
| Hindu and others | Ref. | Ref. |
| Muslim | 0.80*** (0.72, 0.88) | 0.75*** (0.63, 0.90) |
| **Caregiver’s age** |  |  |
| <25 years | Ref. | Ref. |
| ≥25 years | 1.01 (0.94, 1.08) | 0.92 (0.82, 1.04) |
| **Caregiver’s education** |  |  |
| <5 years | Ref. | Ref. |
| ≥5 years | 1.11* (1.01, 1.22) | 1.08* (0.91, 1.29) |
| **Father’s education** |  |  |
| <5 years | Ref. | Ref. |
| ≥5 years | 1.19*** (1.09, 1.29) | 1.20* (1.02, 1.40) |
| **Wealth index** |  |  |
| Poor | Ref. | Ref. |
| Middle | 1.03 (0.94, 1.12) | 1.06 (0.91, 1.23) |
| Rich | 1.12* (1.03, 1.22) | 1.19 (1.02, 1.39) |
| **Morbidity status** |  |  |
| No | Ref. | Ref. |
| Yes | 0.87*** (0.80, 0.95) | 0.93 (0.83, 1.05) |
| **CHW’s visit within the last 12 months** |  |  |
| No | Ref. | Ref. |
| Yes | 1.18*** (1.09, 1.27) | 1.10 (0.95, 1.27) |
| **Effective coverage of MNP** |  |  |
| No | Ref. | Ref. |
| Yes | 1.13* (0.91, 1.35) | 1.30** (1.12, 1.51) |

*p <0.05, ** p<0.01, ***p<0.001

**Table S6** General Guideline for Qualitative Data Collection (Service Providers)

| **Ba Background information** | Name, age, education, occupation/designation, monthly income, sources of income, working area, work experience, mobile number |  |
| --- | --- | --- |
| Discussion Area | Key questions & Probes | Respondents |
| Job responsibilities | Please describe your job responsibilities. | SS, SK, PO, FO, UM, DM |
| Involvement with the MIYCN program | When and how did you get involved with MIYCN program implementation activities?  Is it possible/feasible for you to implement program activities on a regular basis? If yes/no, why? |  |
| Training received since joining BRAC | Did you receive any sort of training after joining BRAC? If yes, please probe: title, topics covered, facilitators, duration of training etc. If no, then why? |  |
| Training received on Pushtikona | Did you receive any training on Pushtikona? If yes, please probe: title, topics covered, facilitators, duration of training etc.  Please tell me about the logistics and materials you used received in the training; can you show me some training materials? If you didn’t receive any materials during/after training, please tell us the reason.  Did you understand the topic discussed? If no, what were the challenges you faced? | SS, SK, PO, FO |
| Effectiveness of the training | How were you able to utilize the knowledge from the training Pushtikona during field implementation? Please explain.  Did you work on Pushtikona before receiving the training? How? (Please explain challenges/barriers).  Please tell me your opinions about the training on Pushtikona (probe: good/ worst thing about the training/training room, session, facilitator, language, MIYCN register, reporting format, monthly sales target, etc.) | SS, SK, PO, FO |
| Role of supervisors in increasing knowledge/skill of SSs/PSs | What does your supervisor do to improve your knowledge and skills? | SS, SK, PO, FO, UM |
| Visits & provision of messages on Pushtikona | What are your roles and responsibilities in regards to Pushtikona program implementation? How often do you visit the field/HHs to sell Pushtikona?  How do you counsel mothers/caregivers about Pustikona? What are the field challenges related to counselling? |  |
| Opinions on Pushtikona messages | *Pushtikona enhances food values if it is mixed with family foods.* To what extent do you agree or disagree with this statement? What is your opinion on this? | SS, SK |
| Pushtikona sales and afterwards HH visit and follow up | How do you follow up with a HH after selling Pushtikona? How and when?  What are the issues that motivate/de- motivate you from revisiting the HH after selling Pushtikona? | SS, SK, PO, FO |
| Information on Pushtikona sales | How do you sell Pushtikona? (please explain sales pattern, e.g. sachet/box/during HH visits)  Are you able to sell Pushtikona equally per month? If not, then why?  What are the challenges you observed during sales of Pushtikona? How do you overcome these issues? | SS, SK |
| Provision of Incentive | What is your overall opinion about the provision of incentives? (Probe: amount/timely disbursement/rigorous monitoring).  Did you ever receive any incentives on Pushtikona? If yes, please give details (when, how and why you receive, how much, activities done to receive incentives, by whom).  Did you receive incentive for other activities as well? If yes, please give details (when, how and why you receive, how much, activities done to receive incentives, by whom). | SS |
| Supportive supervision and monitoring | Does anyone from BRAC visit you to monitor your MIYCN implementation activities? Probe: who/when/frequency/instruction/advice). Were those visits helpful? | SS, SK, PO, FO |
| Field challenges and their solutions | What do you do if you face any challenges during field visits? (Probe: Pushtikona selling, counselling, irregular consumption, return of Pushtikona). How do you inform your supervisor on these issues? Please explain. | SS, SK, PO, FO |
| Supply and distribution of Pushtikona | What is the process of supply and distribution of pustikona? Did you face any challenges in this process? | SS, SK, PO, FO |
| Activities taken to raise awareness | Do you participate in any activities BRAC arranges to ensure awareness on Pushtikona? If yes, please explain. Were there any challenges? | SS, SK, PO, FO |

**Table S7** General Guideline for Qualitative Data Collection (Caregivers)

| **B Background information:** | Name, age, education, occupation, religion, household income  (monthly), number of under-five children, husband’s occupation, decision making process of the household, mobile number |
| --- | --- |
| Discussion Area | Key questions & Probes |
| Feeding practices of a children | What do you feed to your child? What is your opinion about this feeding practice? How long you are feeding this to your child.  Do you think your child needs separate food preparation? Can you afford those?  Did you receive any information about the age specific feeding practices for your children? If yes, from whom and what you have learned? Do you follow those suggestions? |
| Interaction between SS and caregivers regarding Pushtikona | Did you receive any information from anyone about pushtikona? If yes, when and what did you learn? Did you follow those suggestions? If no, why? Please explain.  How do you get poushtikona? Did you ever feed pushtikona to your children? Did you face any difficulties in feeding pushtikona? Did your child have any adverse effect after feeding Pushtikona?  Did your child like the taste of the food after fortified with the pushtikona? If no, what did you do? |
| Ensuring compliance of feeding pushtikona | How the SS helped you in feeding pushtikona regularly? How the SS solve the problem if your child does not like the taste of the food fortified with pushtikona? |
| Recommend pushtikona to others | Do you ever recommend feeding pushtikona to other mothers/caregivers?  What is the benefit of feeding pushtikona to the children you think? |
| Suggestion for improved counselling | How well a SS can convince a mother/caregiver about regular purchasing and feeding of pushtikona to their children? Can you please demonstrate us? |
